# Late dysphagia after changes in high-dose clinical tumour volume margin for head and neck cancer patients

**DOI:** 10.2340/1651-226X.2025.43924

**Published:** 2025-09-18

**Authors:** Ruta Zukauskaite, Jesper Grau Eriksen, Jørgen Johansen, Eva Samsøe, Morten Horsholt Kristensen, Lars Johnsen, Camilla Kjaer Lonkvist, Cai Grau, Jens Overgaard, Christian Rønn Hansen

**Affiliations:** a Department of Oncology, Odense University Hospital, Odense, Denmark; b Department of Experimental Clinical Oncology, Aarhus University Hospital, Aarhus, Denmark; c Department of Oncology, Zealand University Hospital, Næstved, Denmark; d Laboratory of Radiation Physics, Odense University Hospital, Odense, Denmark; e Department of Oncology, Herlev and Gentofte Hospital, University of Copenhagen, Herlev, Denmark; f Danish Centre for Particle Therapy, Aarhus University Hospital, Aarhus, Denmark

**Keywords:** Head and neck, late toxicity, dysphagia, CTV margin, guidelines

## Abstract

**Background and purpose:**

One of the factors influencing disease control and toxicity risk after radiotherapy is selection of treatment volume margin. This study evaluates whether different gross tumour volume (GTV) to high-dose clinical target volume (CTV1) margins impact dysphagia in a cohort of head and neck squamous cell carcinoma (SCC) patients.

**Patient/material and methods:**

Data of patients receiving primary IMRT-based radiotherapy for SCC for the oropharynx, hypopharynx, and larynx at three treatment centres between 2010 and 2015 were retrospectively collected. Treatment planning followed two DAHANCA guideline periods: pre-2013 (varying GTV-CTV1 margins), and post-2013 (isotropic 5 mm margin). Treatment plans were collected for 1,913 patients. GTV–CTV1 margins were calculated as median surface distance from GTV to CTV1. Dysphagia was graded using modified DAHANCA ordinal scale. For each patient, the highest score of dysphagia during 5-year follow-up period was chosen for analysis.

**Results:**

Dysphagia data were available for 1,706 patients (89%). The median GTV–CTV1 margin was 9.0 mm in 2010–2012 and 4.7 mm in 2013–2015. The severity of dysphagia was more pronounced in patients treated during 2010–2012 (p = 0.003). Predictors of grade ≥ 2 dysphagia included larger GTV (odds ratio [OR]: 1.7; p < 0.001), larger GTV–CTV1 margin (odds ratio [OR] of 1.3 per cm; p = 0.04), and tumour localisation other than oropharyngeal p16+carcinomas (p = 0.002). Male sex, non/previous smoking status, and application of chemotherapy were associated with less severe dysphagia.

**Interpretation:**

Tumour volume and GTV–CTV1 margin are dominant geometric parameters influencing dysphagia risk following curative radiotherapy

## Introduction

Primary radiotherapy (RT) is the foremost treatment modality for squamous cell carcinomas originating in the pharynx and larynx in Denmark. Loco-regional (LR) control is increasing, mainly for patients with favourable disease-related factors [1].

Despite advancements in treatment planning and delivery that have improved disease control, intensity modulated radiotherapy (IMRT) continues to carry a significant risk of both acute and late toxicities [2–6]. Even with precise targeting, irradiation of organs at risk near high-dose treatment areas often leads to considerable acute toxicities, such as dermatitis, mucositis, oedema, and late toxicities, including dysphagia and xerostomia. These side effects can significantly compromise patients’ quality of life (QoL).

A potential contributing factor to the development of side effects may be the extent of treatment volumes defined by the clinicians. A primary step in good treatment planning is optimal clinical target definition based on gross tumour volume (GTV) identification, followed by careful IMRT planning [7]. That allows reduction in treatment volume compared to 3D techniques and makes it possible to make a more conformal dose distribution in general [8, 9]. During the early adoption of IMRT, concerns arose that the more conformal dose distribution might compromise local control. To mitigate this risk, relatively large clinical target volume (CTV) margins accounted for potential microscopic spread. This cautious approach, while aiming to preserve LR control, may have inadvertently contributed to larger treatment volumes and, consequently, increased risk of toxicity. After general implementation of IMRT, the margins varied considerably, often using at least 1 cm for high-dose CTV volume (CTV1) [8]. No randomised trial comparing treatment outcomes using different margins around GTV to form CTV have been performed.

Over the last decade, an increasing number of retrospective studies have shown that local and or LR recurrences after primary curative RT emerge mainly in the GTV, irrespective of the magnitude of the GTV–CTV1 margin [10–13]. Furthermore, recent publications demonstrate a significant impact of reduced margins on toxicities while maintaining LR control [14–16].

Since the introduction of IMRT techniques in Denmark around 2004, modifications in GTV–CTV1 margins were subsequently implemented [8, 11, 17]. Up to 2013, margins varied among centres. Since 2013, a standardised 5-mm margin around the GTV for generating CTV1 has been used in all head and neck cancer centres in Denmark, and adopted internationally in 2018 [11, 18]. The current study aimed to evaluate the impact of different GTV to high-dose CTV (CTV1) margins, corrected for other clinical and treatment-related factors, on late dysphagia after curative RT for consecutively treated patients with head and neck squamous cell carcinomas (HNSCC).

## Patients/material and methods

This cohort study included consecutive patients receiving definitive IMRT-based treatment for loco-regionally advanced oropharyngeal, hypopharyngeal, or laryngeal squamous cell carcinomas in three national HN cancer centres. The study population consisted of patients treated 3 years before (2010–2012) and 3 years after (2013–2015) implementing the 2013 DAHANCA (Danish Head and Neck Cancer Study Group) RT guidelines [19], and with a complete follow-up of 5 years as described recently [11]. Data were obtained on 25 October 2024.

The treatment was guided by DAHANCA criteria, with either (1) accelerated hyperfractionated RT with prescribed dose 76–66–56 Gy to CTV1–CTV2–CTV3, respectively in 56 fractions (fr), 10 fr/w (*n* = 62 for the 2010–2012 time period, and *n* = 95 for the 2013–2015 time period); (2) accelerated RT with prescribed dose 66–60–50 Gy in 33 fr, 6 fr/w (*n* = 761 for the 2010–2012 time period, and *n* = 862 for the 2013–2015 time period); or (3) conventional RT regime with prescribed dose 66–60–56 Gy, 33 fr, 5 fr/w (*n* = 73 for 2010–2012 time period, and *n* = 61 for the 2013–2015 time period). If primary or nodal GTV exceeded 4 cm, the prescription doses for CTV1 were 78, 68, and 68 Gy, respectively in the three regimes. The radiosensitiser nimorazole and platinum-based chemotherapy were added if indicated [11]. During treatment planning either planning CT-MRI or planning CT registered with diagnostic PET-CT (positron emission tomography-computed tomography) or MRI (magnetic resonance imaging) were performed. In the current study, all DICOM treatment plans were retrospectively collected in the national treatment plan bank, DcmCollab [20], and the GTV–CTV1 margins (in millimetres) were quantitatively assessed by calculating the median surface distance from the primary GTV to CTV1. This was performed by measuring the shortest distance from each point on the GTV surface to the surface of CTV1, and taking the median value for analyses [8, 21]. High-dose CTV–PTV margin was 5 mm.

### Assessment of late dysphagia

Patients underwent regular follow-up assessments every 4 months during the first 2 years (before 2014) or every 6 months during the first 2 years (after 2014), and at a minimum annually from year 3–5 [3, 11]. Dysphagia was scored by the physician, guided by the patients’ description of daily eating experiences. A modified Late Effects on Normal Tissues – Subjective Objective Management Analytic (LENT–SOMA) scale for late toxicities was used: 0: no dysphagia; 1: symptomatic, but able to eat regular diet; 2: symptomatic and altered eating/swallowing, soft food; 3: symptomatic and altered eating/swallowing, only fluid food; 4: severely altered eating/swallowing; tube feeding or hospitalisation; urgent intervention indicated. The follow-up protocol typically involved flexible endoscopy and clinical examination as described previously [11]. The current study endpoint, dysphagia, was recorded from the sixth month of follow-up until the completion of follow-up at 5 years.

The highest physician-rated dysphagia score was chosen for analysis of individual patients during a 5-year follow-up. The dysphagia scores were dichotomised into 0–1 (non or minimally altered daily nutrition) and 2–4 (altered eating) for further analyses.

### Statistical analysis

Dysphagia was dichotomised for the binomial logistic regression to identify predictors influencing pronounced dysphagia, including sex, smoking status, tumour site, RT dose, GTV volume (log-transformed), GTV–CTV1 margin, use of radiosensitiser and chemotherapy. The parameters were chosen based on general prognostic factors influencing morbidities. Besides investigating the highest dysphagia score, logistic regression analysis was also performed for dysphagia scores at 6, 12, and 24 months.

The 2010–2012 cohort with non-standardised (0–10 mm) GTV–CTV1 margins was compared with the 2013–2015 cohort where a standardised 5-mm margin was used. The relationship between patient variables was examined using Pearson’s Chi-square or Fisher’s exact test. All tests were two-tailed. A *p* < 0.05 was considered significant. Statistical analyses were done using IBM SPSS^®^ Statistics version 23.

## Results

All patients with complete DICOM treatment plans available were included (*n* = 1,913). Dysphagia data were available for 1706 (89%) of the cohort. The patient and disease characteristics for patients with available dysphagia scoring are illustrated in Table 1.

**Table 1:** Patient and disease characteristics in the treatment period 2013–2012 and 2013–2015 (only for the patients with available dysphagia data).

	2010–2012 (*n* = 798) (%)	2013–2015 (*n* = 908) (%)	*p*
**Sex**			
Male	602 (75.4)	693 (76.3)	0.7
Female	196 (24.6)	215 (23.7)	
Age (median)	61	63	0.2
**Smoking:**			
Never/previous/ unknown	496 (62.2)	560 (61.7)	0.8
Current	302 (37.8)	348 (38.3)	
**Primary tumour:**			
Larynx	199 (24.9)	200 (22.0)	0.07
Oropharynx p16+	337 (42.2)	434 (47.8)	
Oropharynx p16- /p16 unknown	180 (22.6)	174 (19.2)	
Hypopharynx	82 (10.3)	100 (11.0)	
**T classification:**			
1–2	557 (69.8)	671 (73.9)	0.07
3–4	241 (30.2)	237 (26.1)	
**N disease presence:**			
N0	270 (33.8)	285 (31.4)	0.3
N+	528 (66.2)	623 (68.6)	
**Treatment dose (Gy):**			
66–68	744 (93.2)	822 (90.5)	0.05
76–78	54 (6.8)	86 (9.5)	
**Fractionation (fr/week):**			
Conventional (5)	59 (7.4)	50 (5.5)	0.1
Accelerated (6 or 10)	739 (92.6)	858 (94.5)	
**Radiosensitizer**			
Yes	715 (89.6)	859 (94.6)	0.001
No	83 (10.4)	49 (5.4)	
**Chemotherapy**			
Yes	381 (47.7)	471 (51.9)	0.09
No/unknown	417 (52.3)	437 (48.1)	
GTV-CTV1, median (cm)	0.9 (0.0–0.97)	0.47 (0.45–0.53)	< 0.001

**Figure 1:**
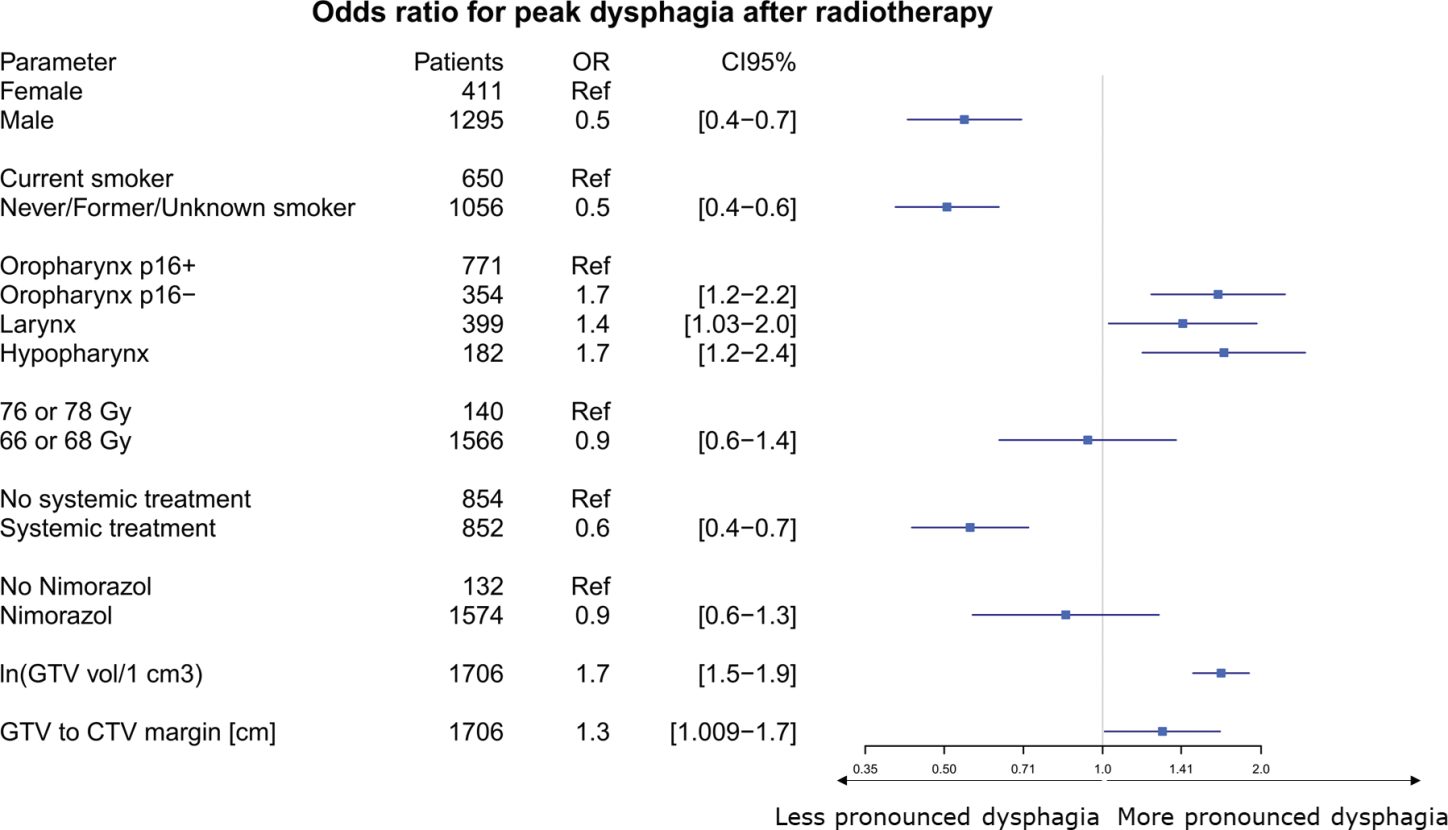
Forest plot of variables associated with the risk of more pronounced dysphagia, displaying odds ratios (OR) with 95% confidence intervals (CI). The vertical line at OR = 1 denotes no effect. An OR greater than 1 indicates an increased risk of higher-grade dysphagia, whereas an OR less than 1 indicates a decreased risk. The reference category for each variable is noted as ‘Ref’.

**Figure 2:**
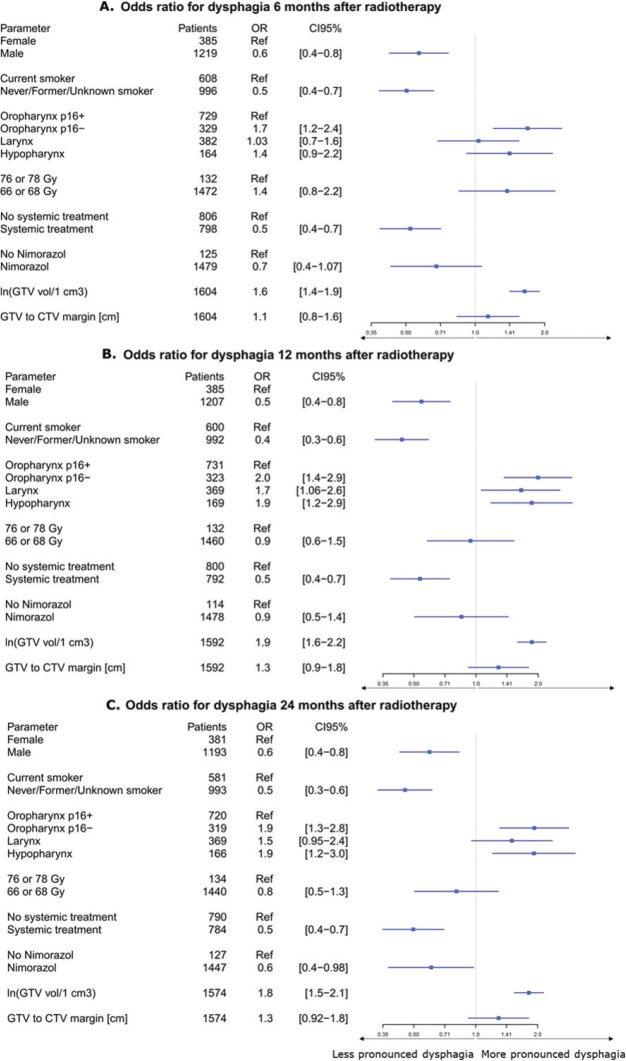
Forest plot of variables associated with the risk of more pronounced dysphagia, for 6 months (A), 12 months (B), 24 months (C), displaying odds ratios (OR) with 95% confidence intervals (CI). The vertical line at OR = 1 denotes no effect. An OR greater than 1 indicates an increased risk of higher-grade dysphagia, whereas an OR less than 1 indicates a decreased risk. The reference category for each variable is noted as ‘Ref’.

In 2010–2012, 298 (37%) patients had dysphagia grade 2+ compared with 278 (31%) in 2013–2015 (*p* = 0.003). Multivariable logistic regression analysis showed that larger GTV volume (OR: 1.7 [95% CI: 1.48–1.89], *p* < 0.001), and GTV–CTV1 margin (OR of 1.299 per cm [95% CI: 1.00–1.67], *p* = 0.04). Patients with carcinomas other than p16+ oropharynx also demonstrated higher grades of dysphagia (*p* = 0.002, comparison with other localisations is shown in Figure 1). In contrast, male sex (OR: 0.55 [95% CI: 0.43–0.70], *p* < 0.001), non-current smoking status (OR: 0.51 (95% confidence interval [CI]: 0.40–0.63), *p* < 0.001), concomitant chemotherapy (OR: 0.56 (95% CI: 0.43–0.72), *p* < 0.001) were predictors to less pronounced dysphagia (Figure 1). Performing similar analyses for dysphagia at 6, 12, and 24 months did not change these findings. In this supplementary analysis GTV–CTV1 margins were not significantly associated with the severity of dysphagia: OR for dysphagia at 6, 12, and 24 months was 1.14 per cm (95% CI: 0.83–1.55, *p* = 0.42), 1.28 per cm (95% CI: 0.91–1.80, *p* = 0.15), and 1.29 per cm (95% CI: 0.92–1.81, *p* = 0.14), respectively (Figure 2. A-C).

## Discussion and conclusion

In the last decades, a significant awareness concerning side effects after curative cancer treatments has risen. Increasing access to different treatment modalities and longer life expectancy leaves an important task of improving the expected QoL for patients with head and neck cancer after comprehensive RT or concomitant chemo-radiotherapy. In the present study, we demonstrate that the reduced GTV–CTV1 margin entails a decrease in physician-scored late dysphagia.

For HNSCC treatment-induced toxicities, the first analyses and comparisons of IMRT to 2D or 3D RT techniques in the early 2000s were mainly aimed at minimising xerostomia [4, 6, 22, 23]. However, an increasing amount of effort to spare organs at risk involved in swallowing emerged soon after [24], and both parotid sparing and swallowing sparing IMRT became a part of treatment planning for HNSCC patients [5, 25, 26]. A review concerning treatment outcomes and late toxicities using IMRT up to 2018 have highlighted various diversities in the studies related to treatment planning, scoring of toxicities, doses to the tumour, and organs at risk, among others and did not demonstrate strong superiority of IMRT for late dysphagia [27]. Over the past decade, IMRT treatment planning and delivery have advanced significantly. The generation of treatment volumes (clinical target volume (CTV), planning target volume, (PTV)) has become more standardised with continued reliance on GTV identification through available imaging modalities [7]. The margin from GTV to high-dose CTV have become more compact and reduced, but propitiously, an increasing number of analyses show that recurrences mainly occur in the GTV despite the size of the margin [8, 10–12]. Since 2019, publications have appeared from researchers in the Netherlands showing that margin reduction results in maintained LR control rates and reduced toxicities [14–16]. After GTV–CTV1 margin reduction from 10 (*n* = 155, 2015–2017 period) to 6 mm (*n* = 155, 2017–2019 period), grade 2 or more dysphagia was significantly lower in the 6 mm group compared to the 10 mm group, 67% versus 85%, *p* < 0.01 [15]. The multivariate analyses for dysphagia showed that margin (*p* = 0.04) and unilateral elective irradiation (*p* = 0.05) were the only predictors, whereas other disease and treatment factors did not significantly influence dysphagia [16]. In contrast, our results for 1,706 patients showed that primary GTV volume was the main factor influencing the severity of late dysphagia (*p* < 0.001), followed by GTV–CTV margin (*p* = 0.04). Our data lack an important information concerning elective neck irradiation. Previously published results for this cohort showed a non-significant tendency for a better 3-year local control (0.84 vs. 0.87, *p* = 0.06) after reducing the GTV–CTV1 margin from median 9 to 5 mm [11]; however, presumably more linked to other factors than margin. The modest impact of margin in reduction of dysphagia in present data compared to the Dutch studies may be partly explained by the fact that the initial margins in the Dutch cohort were larger (10 mm), and the final reduced margin remained greater (6 mm) than in our cohort (to 5 mm).

The doses to PCMs (pharyngeal constrictor muscles) and the oral cavity are reported to be the main dosimetric parameters influencing pronounced dysphagia [16, 25, 28, 29]. In Denmark, treatment plan optimisation based on swallowing structures became a standard of treatment around 2015. Likewise, the parallel analyses of current data by Stougaard et. al indicate that higher mean radiation doses to the lower PCM (OR = 1.06 per 5 Gy, *p* < 0.001) and oral cavity (OR = 1.04 per 5 Gy, *p* < 0.001) significantly increased dysphagia risk [30] in line with Dutch data [16].

One unexpected finding was that concomitant chemotherapy had a strong association with less pronounced dysphagia. This confounder might be related to the fact that patients were selected for chemotherapy based on different patient and disease-related factors that may be linked to the risk of dysphagia [3, 31]. As baseline dysphagia was not recorded in the cohort, there could be a higher baseline dysphagia in the patients who did not receive chemotherapy.

Due to increasing treatment possibilities and longer life expectancy, as well as discrepancies among physician-rated and patient-reported toxicities, awareness concerning patient-reported outcomes has gained an important role for patients with HNSCC [5, 32–34]. Different questionnaires, for example, EORTC QLQ-H&N35 and MDADI composite score, can be used. However, no consensus has yet been reached on the simplest patient questionnaire and the most informative one for clinicians. Additionally, big data analysis shows that the most meaningful information can be condensed to less than 30 questions [33]. The patient reported outcomes (PRO) measurements were not a standard part of follow-up in DAHANCA centres during this period. Only physician-rated toxicities were prospectively registered and were the basis for present analyses. The most optimal way to identify the appropriate PRO tool is in a randomised trial. The DAHANCA 35 trial (ClinicalTrials.gov ID NCT04607694), currently recruiting patients to receive either primary IMRT or IMPT for locoregionally advanced HNSCC, applies EORTC QLQ-C30, HN35, MDADI, and EQ 5D for PROs [26, 35]. It is expected that the results of this trial will help construct a compact PRO measurement tool to use in the coming DAHANCA trials. Furthermore, planning to continue follow-up using PROs up to 10 years in DAHANCA 35 will also help to draw up a more realistic picture for late toxicities for patients.

In conclusion, this study demonstrates that GTV to high-dose CTV1 margins was related to more severe physician-rated late dysphagia during the 5-year follow-up after completion of definitive IMRT-based treatment. Based on increasing evidence that the GTV–CTV1 margin does not directly influence LR control, a randomised national margin reduction trial is planned, incorporating physician-rated and patient-reported outcomes for treatment morbidities.

## Data Availability

The data used in this study contain sensitive patient information and are not publicly available due to restrictions imposed by the General Data Protection Regulation (GDPR). Access to the data is limited to authorised researchers through institutional agreements and ethical approvals.
